# Standards-Compliant Multi-Protocol On-Board Unit for the Evaluation of Connected and Automated Mobility Services in Multi-Vendor Environments †

**DOI:** 10.3390/s21062090

**Published:** 2021-03-17

**Authors:** Roshan Sedar, Francisco Vázquez-Gallego, Ramon Casellas, Ricard Vilalta, Raul Muñoz, Rodrigo Silva, Laurent Dizambourg, Antonio Eduardo Fernández Barciela, Xavier Vilajosana, Soumya Kanti Datta, Jérôme Härri, Jesus Alonso-Zarate

**Affiliations:** 1 Centre Tecnològic de Telecomunicacions de Catalunya, 08860 Castelldefels, Spain; francisco.vazquez@cttc.es (F.V.-G.); ramon.casellas@cttc.es (R.C.); ricard.vilalta@cttc.es (R.V.); raul.munoz@cttc.es (R.M.); jesus.alonso@cttc.es (J.A.-Z.); 2 Peugeot Citroën Automobiles, 78943 Velizy-Villacoublay, France; rodrigo.silva1@mpsa.com (R.S.); laurent.dizambourg@mpsa.com (L.D.); antonioeduardo.fernandez@mpsa.com (A.E.F.B.); 3 Worldsensing S.L., 08014 Barcelona, Spain; xvilajosana@worldsensing.com or; 4 Computer Science, Telecommunications and Multimedia Department, Universitat Oberta de Catalunya, 08018 Barcelona, Spain; 5 EURECOM, 06904 Sophia Antipolis, France; Soumya-Kanti.Datta@eurecom.fr (S.K.D.); Jerome.Haerri@eurecom.fr (J.H.)

**Keywords:** 5G, mobile edge computing, V2X communication, cooperative perception, intelligent transport systems

## Abstract

Vehicle-to-everything (V2X) communications enable real-time information exchange between vehicles and infrastructure, which extends the perception range of vehicles beyond the limits of on-board sensors and, thus, facilitating the realisation of cooperative, connected, and automated mobility (CCAM) services that will improve road safety and traffic efficiency. In the context of CCAM, the successful deployments of cooperative intelligent transport system (C-ITS) use cases, with the integration of advanced wireless communication technologies, are effectively leading to make transport safer and more efficient. However, the evaluation of multi-vendor and multi-protocol based CCAM service architectures can become challenging and complex. Additionally, conducting on-demand field trials of such architectures with real vehicles involved is prohibitively expensive and time-consuming. In order to overcome these obstacles, in this paper, we present the development of a standards-compliant experimental vehicular on-board unit (OBU) that supports the integration of multiple V2X protocols from different vendors to communicate with heterogeneous cloud-based services that are offered by several original equipment manufacturers (OEMs). We experimentally demonstrate the functionalities of the OBU in a real-world deployment of a cooperative collision avoidance service infrastructure that is based on edge and cloud servers. In addition, we measure end-to-end application-level latencies of multi-protocol supported V2X information flows to show the effectiveness of interoperability in V2X communications between different vehicle OEMs.

## 1. Introduction

The integration of modern wireless communication technologies into vehicles has paved the way to exchange information between vehicles, between vehicles and other road users, and with an increasingly intelligent and connected road infrastructure [1,2]. Such cooperation between other connected objects has been the foundation in realising cooperative intelligent transport systems (C-ITS) in the context of cooperative, connected, and automated mobility (CCAM). C-ITS is an emerging use case of intelligent transport systems (ITS) [3,4] that enables real-time wireless communication and information sharing between ITS stations, e.g., vehicles or roadside units (RSUs), in order to achieve coordinated decisions through cooperation, thus extending the capabilities of a vehicle beyond the scope of a typical stand-alone entity.

Modern vehicles are progressively turning into sophisticated computing units able to gather, process, and exchange information with other connected objects [5]. They will be typically equipped with ultra-high-definition cameras, radars, LiDARs, ultrasonic range finders, and other types of sensors, which allow for sensing vehicles’ proximity. Additionally, modern vehicles are equipped with an on-board unit (OBU) for V2X communication, which enables vehicles to effectively exchange locally sensed data with the other external entities over wireless connectivity. Such V2X-enabled vehicles are capable of sharing information over vehicle-to-vehicle (V2V), vehicle-to-infrastructure (V2I), vehicle-to-pedestrians (V2P), and vehicle-to-network (V2N) communications. V2X connectivity extends the perception range beyond the limits of on-board sensors of a vehicle, thus supporting emerging vehicular services that will increase traffic efficiency and road safety.

With the emergence of 5G, the use of cellular V2X (C-V2X) [6,7] is gaining more attention worldwide through the execution of promising trials of vehicular services [8,9]. For example, a mobile edge computing (MEC) infrastructure was deployed in Germany [10] along 30 km of the A9 motorway, where it was demonstrated that the vehicles can exchange information with an end-to-end latency below 20 ms using LTE. Similarly, but in a different context, the on-going 5G Cross-Border Control (5GCroCo) project [11] is conducting trials of C-V2X based vehicular use cases that are deployed in cross-border scenarios. One of the use cases deployed in 5GCroCo is the anticipated cooperative collision avoidance (ACCA) service [12]. The aim of the ACCA service is to anticipate impending collisions ahead of time and, subsequently, inform approaching vehicles to avoid any dangerous event, e.g., stationary vehicle, an accident, a vehicle with emergency brake lights, or a traffic jam, among others. As such, each vehicle shares the status information and detected roadside hazardous events with the ACCA service deployed on the MEC infrastructure, which, in turn, creates a distributed dynamic map with all of the collected information and informs those vehicles that are required to include all the relevant information within their geographical region of interest (ROI).

One of the challenges in the deployment of the ACCA service is to facilitate seamless interoperability among OBUs from different original equipment manufacturers (OEMs), since they typically need to support different V2X protocols to communicate with heterogeneous backend applications that are allocated on the MEC/cloud servers. In the cross-border field trials and development of ACCA in the 5GCroCo project, two OEMs are involved: Peugeot Citroën Automobiles (PSA) and Renault, S.A. (RSA). As a result, the testing of the ACCA functionalities and the interoperability of V2X communication interfaces can be a complex and expensive task, as this requires the use of several commercial OBUs from each OEM.

Being motivated by this need, we present in this paper an experimental OBU that supports two V2X communication protocols to exchange information with applications running on the MEC/cloud infrastructure: UDP/IP protocol, carrying ETSI ITS ASN.1 encoded messages; and, the MQTT protocol on top of TCP/IP, carrying ETSI ITS ASN.1 encoded messages in JSON format. The OBU can readily be integrated into any vehicle and it can also be used as a vehicle emulator in case no real vehicles are in-place. The OBU assists the driver by displaying warnings on a human-machine interface (HMI) upon the detection of hazards on the road as well as adapting own driving intentions upon reception of warnings based on the detected events by other vehicles. It includes an interface to the in-vehicle sensors and actuators network for the acquisition of measurements (e.g., speed of the vehicle) and notifications of detected hazards in real-time. Furthermore, it supports the easy integration of different types of 4G/5G cellular modems for V2N communications.

Note that this paper is an extension of our preliminary work presented as a poster in Sedar et al. [13]. In this paper, we have specifically demonstrated the OBU’s operations in a real-world deployment of the ACCA service components on the MEC and cloud servers [14]. In the experimental evaluation of the OBU’s operations, we have considered three types of V2X information flows (as detailed in Section 5.2) that can occur in a cross-border deployment of the ACCA service. The OBUs established the connectivity with the ACCA service components over LTE cellular technology, and the traffic from the OBUs was generated by emulating vehicle movements in a lab setup (as detailed in Section 5.1). Moreover, we have measured end-to-end application-level latencies for each of those three types of V2X information flows as the key performance indicator, while demonstrating the effectiveness of interoperability in V2X communication between different vehicle OEMs.

The remainder of this paper is organized as follows. In Section 2, we present previous work related to V2X communications technologies and open-source implementations of the ETSI C-ITS protocol stack. Section 3 overviews the architecture and information flow of the ACCA service. Section 4 describes the implementation of the experimental OBU to fulfil the requirements of the ACCA service, including its hardware components, V2X communication protocols, interface with the in-vehicle sensors and actuators network, and software architecture. Section 5 presents the validation of the functionalities and V2X communication interfaces of the OBU, together with performance evaluation results. Section 6 provides insights on the OBU’s effectiveness of interoperability in V2X communication. Finally, Section 7 concludes the paper with proposals for future extensions.

## 2. Related Work

Over the last two decades, several radio access technologies have been proposed in order to cover different aspects of vehicular communications and support the demanding requirements that are posed by V2X applications. In this context, predominantly two radio technologies can be identified for V2X communications: the short-range wireless communication based on the IEEE 802.11p [15,16] and the cellular standards for V2X [17,18] have been developed by the 3GPP.

Within the IEEE 802.11p-based communications, two standards have been developed: ETSI C-ITS in Europe [19,20] and DSRC in North America [21,22]. These radio technologies facilitate the formation of wireless ad-hoc networks whenever vehicles or RSUs are within the coverage range of each other, thus enabling direct communications among vehicles and with the roadside infrastructure.

In the context of ETSI C-ITS, there exist two main open-source software implementations of the ETSI C-ITS protocol stack, namely, OpenC2X [23] and Vanetza [24]. These implementations include the core features and protocols of ETSI C-ITS standard and they are described as follows. The networking and transport layers rely on IP and TCP/UDP for the transmissions of non-safety messages, whereas the GeoNetworking protocol and Basic Transport Protocol (BTP) are used for the transmissions of safety-related messages. The GeoNetworking [25] is an ad-hoc routing protocol designed for multi-hop communications using geographical addressing. The BTP is a connectionless and unreliable packet transport protocol similar to UDP. The facilities layer defines two fundamental message types for the exchange of information between ITS stations, i.e., Cooperative Awareness Message (CAM) [26] and Distributed Environmental Notification Message (DENM) [27]. The CAM is a periodic message that provides status information to neighbouring vehicles and RSUs. Its rate can be varied in-between 1–10 Hz, depending on vehicle dynamics and the congestion of the wireless channel. The DENM is an event-triggered message controlled by the ITS application. Furthermore, both OpenC2X and Vanetza can be configured to work with a commercially available MK5 OBU from Cohda Wireless [28]. The interoperability tests using OpenC2X and Vanetza with MK5 OBU have been successfully validated through the exchange of CAM and DENM messages [29,30].

The C-V2X has been standardised by the 3GPP in Release 14 [7,31]. The lower layers of C-V2X are specified by the 3GPP for radio access, whereas the upper layers (i.e., applications, facilities, networking, transport and security) are reused from the ETSI C-ITS and DSRC standards. This, in turn, allows one-to-one mapping of the existing applications that were already developed using ETSI C-ITS or DSRC and ensures interoperability with the emerging C-V2X ITS applications.

In this work, we have developed an experimental OBU based on OpenC2X. This choice is motivated by the flexibility provided by OpenC2X’s modularised architecture, the existing implementation of a Local Dynamic Map (LDM) for data persistence, and the interface to reading a vehicle’s speed through an On-Board Diagnostics (OBD) adapter. We have extended OpenC2X incorporating several new key software modules that require fulfiling the requirements of the ACCA service described in the next section.

## 3. ACCA Service

In this section, we provide an overview of the ACCA service architecture and the V2X information flow between vehicles and applications allocated outside of the vehicles. It is important noting that this work focuses on the ACCA service architecture designed and implemented in the context of the 5GCroCo project [14].

### 3.1. Architecture

**Figure 1:**
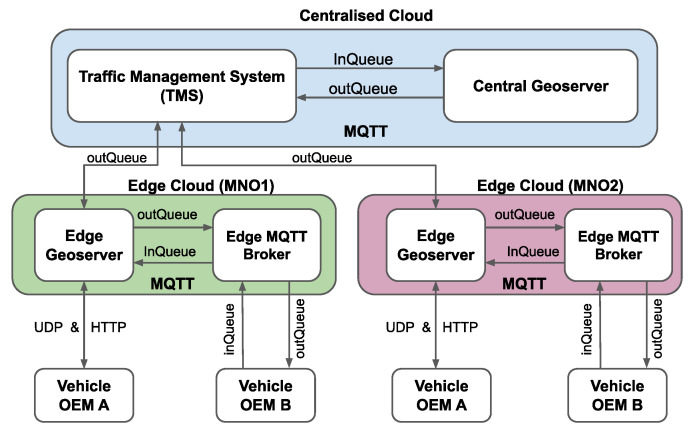
Anticipated cooperative collision avoidance (ACCA) service architecture with two connected vehicle original equipment manufacturers (OEMs). Vehicle OEM A communicates over the UDP/IP and HTTP protocols, and vehicle OEM B communicates over the TCP/IP with MQTT protocol.

Figure 1 shows the high-level architecture of the ACCA service, illustrating the communication interfaces between vehicles and the applications that are deployed outside of the vehicles. For the sake of brevity, we shall refer these applications as backend services hereafter. To achieve the low-latency benefits, the backend services are deployed close to end-users, i.e., at the edge clouds configured on the MEC infrastructure. Moreover, for the sake of simplicity, two edge cloud instances are depicted in Figure 1, where each edge cloud instance is operated by a different mobile network operator (MNO), e.g., MNO1 and MNO2, and these two edge cloud instances are connected to a centralised cloud deployment.

There are two types of vehicles, i.e., OEM A and OEM B, which indicate two different automakers. A vehicle can communicate with the backend service at the edge cloud through an MNO using the preferred V2X communication protocol, i.e., either UDP/HTTP for vehicle OEM A or MQTT for vehicle OEM B, as can be seen in Figure 1. It is obvious that the use of MQTT for V2X communication is a natural choice when the backend services are also based on MQTT. However, the UDP-based communication model is used when transparent hybrid V2X communication (i.e., V2N2V and V2I2V) is expected and end-to-end ETSI ITS [32] security is required.

The backend service deployed at the edge cloud is composed of an Edge Geoserver application and an Edge MQTT broker. The Edge Geoserver basically processes the incoming messages from vehicles and the Edge MQTT broker and, accordingly, aggregates the information. The Edge Geoservers are provisioned to exchange MQTT messages (in JSON format) with the central cloud service hosted in a centralised cloud. The central cloud service is composed of a Traffic Management System (TMS), which is responsible for cross-border message exchange and a Central Geoserver. Similarly, both TMS and Central Geoserver may aggregate, filter, and generally process the incoming messages. TMS and Central Geoserver are both provisioned to exchange MQTT messages with each other, between TMS and Edge Geoservers, and with other external entities through the public Internet. Moreover, two types of message queues, namely, inQueue and outQueue, are utilized to publish/subscribe onto MQTT topics. The inQueue is used to send raw messages that are generated by vehicles towards the TMS and the outQueue offers the dissemination of processed V2X information by the TMS towards the vehicles through the edge cloud services.

It is understood that different MNOs may require different technological interfaces to interconnect with the ACCA service. From the TMS’s perspective, it is possible to expose the application-level information in the form of ETSI ITS messages over different underlying transport protocols. In the presented architecture, MQTT is a convenient option, as it supports geo-tiling of the messages, favouring the subscriptions to only those interested regions that are exposed in the form of MQTT topics, among others. Yet, other technological implementations are also possible. For example, ETSI ITS messages can be transported over HTTP requests, by accessing REST or SOAP APIs that are exposed by the TMS. This imposes different architectural challenges, such as ensuring that the TMS clients periodically poll the services. In some cases, the TMS’s subscribers can be supervisory control and data acquisition (SCADA) systems, mainly being operated by road/infrastructure operators. In this case, and analogously to the REST interface, ETSI ITS messages can be encapsulated over standard OPC/UA services [33], enabling seamless integration to SCADA systems.

### 3.2. V2X Information Flow

There are two phases in the information flow between the different entities of the ACCA service. The first phase performs the authentication and subscription, followed by the exchange of V2X messages in the second phase.

**Figure 2:**
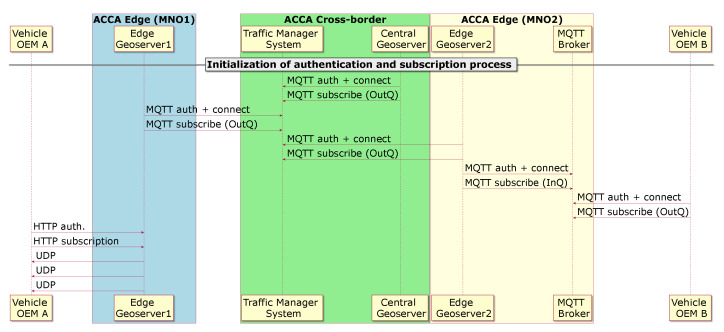
Authentication and subscription process for the connectivity establishment between vehicle on-board unit (OBUs) and the backend services on the edge, and between backend services.

Figure 2 illustrates the authentication and subscription process between backend services, and between vehicles and backend services. Note that there is no specific order in time in the message flows during authentication and subscription. As can be observed, the Edge Geoserver 1, the Edge Geoserver 2, and the Central Geoserver perform the authentication and subscription with the TMS. Additionally, each Edge Geoserver requires the authentication and subscription with the MQTT Broker within its edge cloud. For the sake of simplicity, only the MQTT Broker that is deployed in the ACCA Edge (MNO2) has been included in Figure 2.

Each vehicle performs the authentication and subscription process with the respective backend services. The vehicle OEM A communicates with the Edge Geoserver 1 over HTTP(S) in the process of authentication and subscription and, upon success, it is ready to exchange V2X messages over UDP, as can be observed in Figure 2. Similarly, the vehicle OEM B establishes the connectivity and authenticates with the MQTT Broker of the Edge Geoserver 2, and then it publishes onto inQueue to transmit V2X messages and subscribes to outQueue to receive V2X messages.

**Figure 3:**
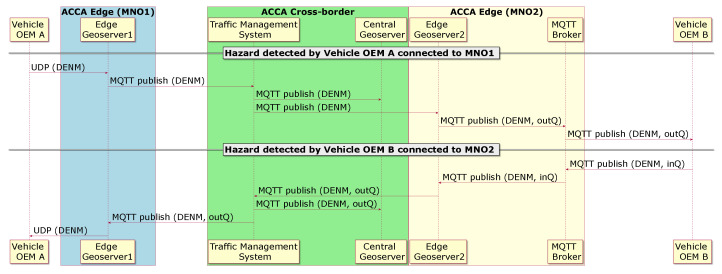
End-to-end propagation of detected hazardous events via Distributed Environmental Notification Message (DENMs) from/to the OBUs from two different vehicle OEMs.

Figure 3 illustrates the way that a hazard information is disseminated within the architecture of the ACCA service when an event is detected by a vehicle. The top left part of the message flow illustrated in Figure 3 shows that vehicle OEM A detects an event and immediately transmits a DENM over UDP towards the Edge Geoserver 1. The Edge Geoserver 1 processes the event information, encodes it into a JSON message and then publishes it into the MQTT Broker and the TMS. Subsequently, the event is reached the Central Geoserver and the Edge Geoserver 2. Finally, the vehicle OEM B receives the event information from the Edge Geoserver 2 through the MQTT Broker deployed on the edge cloud of MNO2.

Similarly, on the bottom right part of the message flow in Figure 3, an event is detected by the vehicle OEM B, which generates a JSON encoded DENM and then publishes onto the MQTT Broker on the edge cloud of MNO2. This information reaches the TMS through the Edge Geoserver 2. Finally, the Edge Geoserver 1 is notified by the TMS and it subsequently transmits over UDP the event information to the vehicle OEM A.

## 4. On-Board Unit Implementation

This section describes the details of the hardware setup, the V2X communication protocols, the interface with the in-vehicle sensors and actuators network, and the software architecture of the experimental OBU that we have designed and implemented in this work.

### 4.1. Hardware Setup

**Figure 4:**
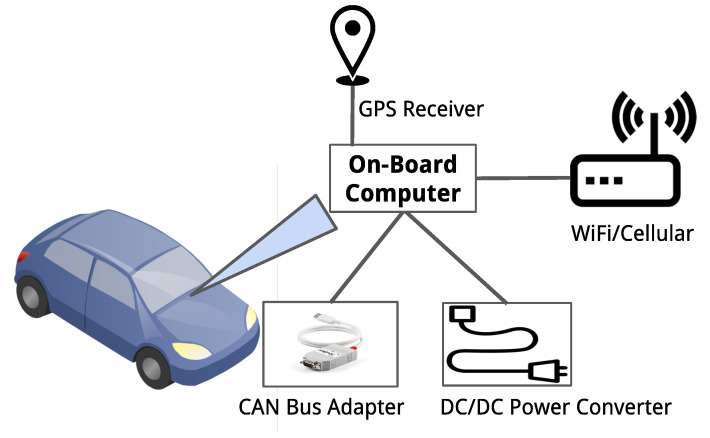
Basic hardware components of the OBU.

Figure 4 shows the hardware components of the experimental OBU. The OBU is composed of a general-purpose computer (i.e., a laptop) and several external hardware devices, as it can be observed. The external hardware devices are a 4G/5G cellular modem, a GPS/GNSS receiver, a Controller Area Network (CAN) bus to USB adapter, and a DC/DC converter. The 4G/5G cellular modem provides V2N connectivity to the outside world. The modem is connected to the computer while using a USB to mPCI adapter. The GPS receiver provides the real-time positioning of the vehicle and, at the same time, the OBU leverages the GPS-PPS signal to run a GPS-based time service while using Network Time Protocol (NTP). In doing so, the OBU keeps the internal clock synchronised with the backend applications of the ACCA service. The CAN bus to USB adapter establishes the connectivity with in-vehicle sensors and actuators network. The DC/DC converter provides the uninterrupted power supply to all pf the components of the OBU.

### 4.2. V2X Communication Protocols

The OBU exchanges two types of ETSI ITS compliant messages with the backend: periodic CAMs and event-triggered DENMs. Towards this, our OBU supports two types of V2X protocols to be compatible with commercial OBUs of vehicles of OEM A and OEM B, respectively, as defined in the ACCA architecture in Section 3.

The first V2X protocol consists of a combination of HTTP and UDP. The HTTP protocol is used for authentication and subscription of the vehicles with the backend, primarily employing POST messages towards the REST API provided by the Edge Geoserver. CAMs and DENMs are encoded according to their respective ASN.1 format [26,27], and they are transmitted as ETSI ITS compliant GeoNetworking messages encapsulated in a UDP packet.

The second V2X protocol is based on MQTT with CAMs and DENMs being encoded in JSON format. MQTT is a lightweight simple messaging protocol that is based on a publish–subscribe mechanism that runs over TCP/IP. MQTT is designed for memory-constrained and low-bandwidth devices and it is extensively used in Internet-of-Things applications. A server named MQTT broker is responsible for the distribution of messages between multiple clients connected to the broker. The information is organized in topics. When a client has a new message to distribute, it publishes the message on a certain topic and the MQTT broker then transmits the message to any clients that are subscribed to that topic.

Topics are hierarchical in nature and resemble a file path. A client may subscribe to topics specifying part of such path as well as using wildcards. In the ACCA service architecture that is depicted in Figure 1, the messages exchanged between different clients are belonging to either inQueue topic or outQueue topic roots. The inQueue topics (e.g., inQueue/v2x/denm/source_id/roi) are used to publish messages from OBUs towards the Edge Geoservers, and from the Edge Geoservers towards the TMS. The outQueue topics (e.g., outQueue/v2x/denm/source_id/roi) are used to publish messages from the TMS towards the Edge Geoservers and from the Edge Geoservers towards the OBUs. We leverage the Quadkeys [34] based approach to represent ROIs as hierarchical topics, commonly referred to as hierarchical binning. In a nutshell, each key is represented a unique tile in the QuadTree geo-tiling grid, in which the grid can recursively be divided into four equal parts until it reaches the threshold number of events in a tile.

It is worth noting that the information distributed over MQTT is ETSI ITS fully compliant CAM and DENM messages, which include the same information as the ASN.1 encoding but in JSON format. A CAM message in JSON format encompasses a header with metadata about the message, including protocol version, station and message identifiers and a basic container object with station type, geographic coordinates (longitude, latitude, altitude) values and confidences. A high frequency container includes information regarding the heading, speed, driving direction, and dimensions of the vehicle. A low frequency container may include additional information like lights or path history and trajectories. A DENM message in JSON format contains a management container with an originating station id, and data about a given event (location and information about the event, such as its geographic location, validity duration, and transmission interval). Optional containers (situation, location) provide information regarding the speed and heading and a trace history. Listing 1 shows an extract of a such JSON encoded ETSI ITS fully compliant ASN.1 DENM message.

Listing 1 **ETSI ITS fully compliant ASN.1 encoded DENM payload in JSON format.**

{  "type": "denm",
 "origin": "on_board_application",
 "version": "1.0.0",
 "source_id": "obu_cttc_1",
 "timestamp": 1574778515425,
 "message": {
  "protocol_version": 1,
  "station_id": 225,
  "management_container": {
   "action_id": {
    "originating_station_id": 41,
    "sequence_number": 1 },
   "detection_time": 503253332000,
   "reference_time": 503253330000,
   "termination": 1,
   "event_position": {
    "latitude": 486263556,
    "longitude": 224921234,
    "altitude": 20000 } } },
...
  "situation_container": {
   "information_quality": 1,
   "event_type": {
    "cause": 97,
    "subcause": 0 },
   "linked_cause": {
    "cause": 1,
    "subcause": 1 } },

### 4.3. Hardware Interfaces with Vehicle’s Sensors and Actuators

There are two possible ways of interfacing our experimental OBU with the in-vehicle sensors and actuators network. One approach is to use the standard OBD interface that is available in all types of vehicles. The OBD interface allows for reading several physical parameters of the vehicle (e.g., speed, motor number of revolutions, etc.), and it is a valuable source of information for the basic diagnosis in mechanical and electronic maintenance.

The second approach is to interface directly with the CAN bus of the vehicle, where all sensors, actuators, and Electronic Control Units (ECU) of the Advanced Driver-Assistance System (ADAS) are connected. However, this approach is not possible with commercial vehicles due to safety and security aspects. In this work, we have integrated our experimental OBU into a prototype vehicle provided by PSA. The prototype vehicle is a DS7 car, which includes a CAN gateway that was developed by PSA. The CAN gateway acts as the bridge that filters and forwards CAN frames between the CAN bus and the OBU. The OBU is equipped with a CAN to USB adapter. The CAN gateway periodically transmits CAN frames every 50 ms that contain sensors’ readings data, such as the speed of the vehicle (km/h) and status information of brakes, emergency brake lights, hazard detection, etc.

**Figure 5:**
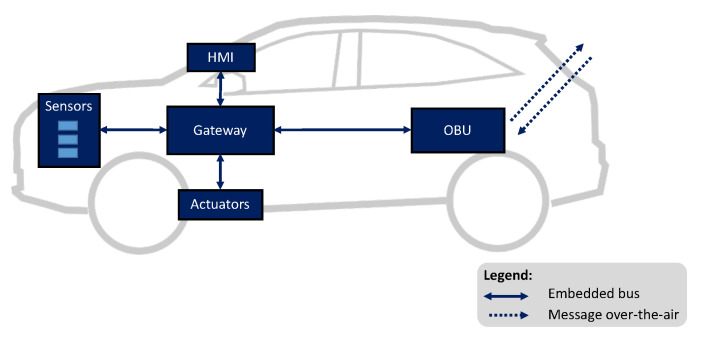
Overview of DS7 vehicle’s embedded system.

Figure 5 depicts an overview of the vehicle’s embedded system. The communication between the vehicle’s CAN gateway and the OBU is realised through a PCAN-USB interface. The vehicle is equipped with on-board sensors, like radars, cameras, or accelerometers, which are capable of sensing the environment of the vehicle. The vehicle’s CAN gateway receives data from sensors and interprets such data as hazard events or only road perception before transmitting them to the OBU. Moreover, the OBU is responsible for receiving V2X messages from the network infrastructure and backend services, and send them to the vehicle core system. An HMI in the car dashboard enables the vehicle to display messages from sensors, as well as events that were received from the backend services. If the reported event leads to a risk of collision, the vehicle core system triggers embedded actuators, adapting its longitudinal behaviour accordingly.

### 4.4. Software Architecture

**Figure 6:**
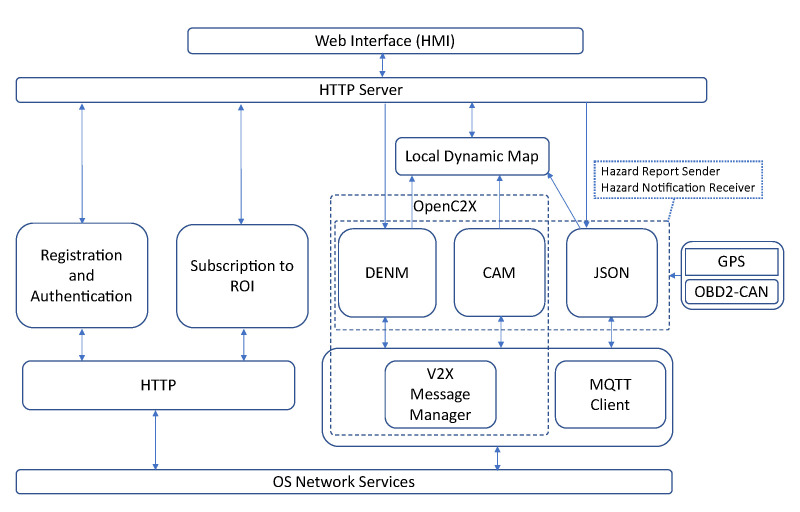
Software architecture of the OBU including the core modules of ETSI C-ITS stack, and other components to support UDP/HTTP and MQTT communications.

The software architecture of the experimental OBU shows in Figure 6. As depicted in the figure, the software architecture consists of decoupled modules that communicate with each other using the asynchronous ZeroMQ messaging library. In what follows, we describe the core functionalities of the OBU software modules.

**Web Interface:** This module provides the GUI that operates as the HMI of the OBU, facilitating the interaction between the driver and the OBU. Through the Web Interface controllers, the driver is able to: (i) select the V2X protocol to communicate with the backend service (UDP/HTTP or MQTT); (ii) initiate the registration and authentication process with the backend service; (iii) configure the subscription parameters to an ROI; (iv) initiate a new subscription to an ROI or a sequence of periodic subscriptions; and, (v) emulate the detection of a road hazard by triggering the transmission of a DENM towards the backend service. In addition, the Web Interface shows the position of the vehicle on a map based on OpenStreetMap, including the locations of road hazards and other vehicles. Furthermore, the Web Interface displays warning messages when the OBU receives DENMs about roadside incidents.

**HTTP Server:** The HTTP server module acts as a middleware between the GUI and the rest of the modules of the OBU. The HTTP server implements a lightweight RESTful web service that translates the GUI-triggered REST commands into ZeroMQ compatible format and then forwards them to the target module. As can be seen in Figure 6, the HTTP server interacts with: (i) the Registration and Authentication module, (ii) the Subscription module, (iii) the DENM module, to trigger the transmission of a new DENM associated to an emulated road hazard, and (iv) the LDM module, to query the positions of the vehicle, road hazards, and other vehicles.

**Registration and Authentication:** This module leverages HTTP(S) in order to register and authenticate the OBU in the backend service. This module is only used when the OBU operates in UDP and HTTP communication mode. The module implements an HTTP(S) client to generate REST commands that contain the credentials of the vehicle. In response, the backend service sends an authentication JSON Web Token (JWT), which is stored on the OBU for subsequent requests, and it is used to verify the authenticity of the OBU by the backend. Section 3.2 describes the process of registration and authentication.

**Subscription to ROI:** This module configures the subscription parameters to an ROI through the Web Interface, and generates requests over HTTP POST. Upon change of an ROI, a new subscription is triggered towards the backend services or when the current subscription expires. The subscription parameters are the geographical coordinates and the area of the ROI within a certain radius, the reporting mode of the hazardous events occurred in the ROI, the type and freshness of the events based on time, and the duration of the subscription. Two approaches are employed by the backend to disseminate information to the vehicles. One approach is the request-response system where a vehicle can query at a time a specific event or all the events that occurred in the ROI. Alternatively vehicles can opt for an uninterrupted connectivity with the backend over a period of time to retrieve information as they occur in the ROI.

**Local Dynamic Map (LDM):** This implements the connectivity to an SQL database, which stores the relevant information extracted from incoming CAM and DENM messages. In addition, the information of CAM and DENM are purged from the database once a pre-defined validity period is expired. The validity period of the information is defined based on the vehicular application requirements.

**Global Positioning System (GPS):** An external GPS receiver is used to acquire the geographical coordinates of the vehicle (i.e., latitude, longitude, and altitude) and then forwards these information to the CAM, DENM, and JSON/MQTT Client modules.

**OBD2-CAN:** The OBD2-CAN module implements the connectivity to the in-vehicle sensors and actuators network either through an OBD or a CAN interface, as described in Section 4.3. Hence, the OBD2-CAN module is configured at run-time based on the availability of either OBD2 or CAN interface. The module retrieves the speed and acceleration of the vehicle and subsequently feeds these data into the CAM and DENM Services. If the module is configured to the CAN bus, then it can retrieve the detected events by on-board smart sensors for collision avoidance. Upon the detection of a road hazard, the OBD2-CAN module notifies the DENM service to trigger the transmission of a DENM.

**DENM Service:** The DENM module is responsible for encoding and decoding DENM messages. The DENMs are event-triggered and can be generated upon the detection of road hazards as well as emulated though the Web Interface. The module fills the fields of DENM message, including geographical coordinates of the hazard and other in-vehicle sensory information provided by GPS and OBD2-CAN modules, respectively, as defined by the ETSI ITS specification [27]. The DENM message is passed to the V2X Message Manager to add the GeoNetworking and BTP headers. Upon reception of DENMs from the backend service, the DENM module extracts the GeoNetworking and BTP headers, decodes the fields of the DENM message, and forwards the relevant information of the event (type, position) to the LDM module.

**CAM Service:** The CAM module is responsible for encoding and decoding CAM messages. The CAMs are generated periodically and included vehicle’s dynamic parameters, attributes, location information, etc. The position of the vehicle is provided by the GPS module and the speed is provided by the OBD2-CAN module. The module fills the fields of CAM message at the facilities layer, as defined by the ETSI ITS specification [26], and passes it to the V2X Message Manager module where the GeoNetworking and BTP headers are added. The module also receives CAMs from the V2X Message Manager that are transmitted by other vehicles, extracts the relevant data, and forwards these data to the LDM module.

**V2X Message Manager:** The V2X Message Manager module implements UDP-based communication interface between CAM and DENM modules and the backend service. The module inserts ETSI ITS compliant GeoNetworking and BTP headers into ASN.1 encoded CAM/DENM messages, encapsulates these messages into UDP packets, and then sends them in unicast to a UDP server allocated in the backend. Upon reception of UDP packets from the backend, the module decapsulates CAM and DENM messages, extracts the GeoNetworking and BTP headers, and forwards CAMs and DENMs to the respective CAM and DENM modules.

**JSON and MQTT Client:** These two modules operate in concert upon selection of the MQTT-based communication mode. This, in turn, provides the seamless interoperability to establish connectivity with backend services, such as centralised traffic management systems or other vehicular services deployed outside of the edge cloud infrastructure, which are accessible through the public Internet. Importantly, these services may not be offered within the ACCA service, but operating as third-party services, e.g., a regional or a countrywide traffic management systems. The MQTT client implementation leverages the JSON encoding functionality to encode CAM and DENM messages and exchange with the interested parties over the MQTT protocol. We employ a direct translation of ASN.1 formatted CAM and DENM objects into JSON encoded messages. The connectivity between the OBU and outside is established through an MQTT broker, onto which the MQTT client subscribes at the inception. Over the MQTT client, the OBU is able to subscribe to several topics that represent geographical regions, as was described in Section 4.2. Thus, the OBU is being notified by the MQTT broker upon receiving fresh events from other connected entities, such as backend services, vehicles, etc.

## 5. Experimental Evaluation

In this section, we present the validation of the functionalities and V2X communication interfaces of the OBU, and we measure the application-level latency as the key performance indicator of the ACCA service.

### 5.1. Test Setup

The test setup is composed of two experimental OBUs, two Edge Geoserver applications, one TMS, and two Edge MQTT brokers that are based on the open-source Eclipse Mosquitto implementation. Each OBU is based on a laptop running Ubuntu 18.04 and connected to an LTE AirPrime EM7565 modem from Sierra Wireless which provides cellular connectivity for V2N communications. The OBUs are connected to the 4G cellular network of Vodafone-Spain.

No real vehicles are involved in this test, as it was performed in a lab environment; therefore, the OBUs are configured to operate as virtual vehicles, where their movements and the detection of hazards are emulated. In this mode, vehicles move based on pre-defined traffic trajectories that are provided from a file containing a sequence of GPS coordinates and associated time-stamps. The Web Interface of the OBUs allows to choose a traffic trajectory file and to start and stop the movements of the virtual vehicle. In each OBU, the CAM messages are periodically transmitted at every 100 ms following the ETSI ITS standard. The DENM messages are also periodically triggered to collect application-level latency measurements for the events that are exchanged between two OBUs and between two OBUs and the TMS. The periodicity is explained in the corresponding test cases.

### 5.2. Functional Validation

**Figure 7:**
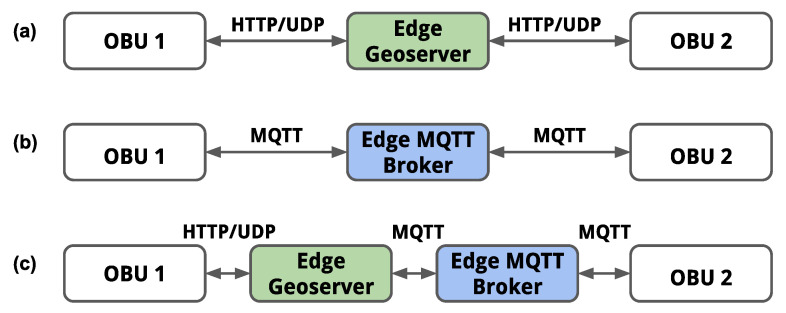
The representation of the test cases with two experimental OBUs. Each test case consists of a different combination of V2X communication protocols between both OBUs and the edge cloud. (**a**) UDP/HTTP-based communications; (**b**) MQTT-based communications; (**c**) Hybrid UDP/HTTP and MQTT-based communications.

We have considered three different test cases to validate the functionalities and V2X communication interfaces of the OBU. For each test case, we have used a different combination of V2X communication protocols between the OBUs connected with one edge cloud, which consists of an Edge Geoserver and an Edge MQTT broker, as depicted in Figure 1. The schematic representation of the test cases is shown in Figure 7 and the operation of the OBUs in each test case is described below.

#### 5.2.1. UDP/HTTP-Based Communications

In this test case, both OBUs (1 and 2) communicate with the Edge Geoserver using UDP/HTTP protocol. At inception, OBU 1 and OBU 2 are registered in the Edge Geoserver and then mutually authenticated over HTTP(S). Subsequently, both OBUs can subscribe to an ROI that is based on the current geographical region. Subsequently, from the web interface of OBU 1, we trigger the transmission of a DENM message towards the Edge Geoserver (as a unicast UDP packet) to indicate an emulated hazardous event detection within the current virtual location of OBU 1. Consequently, the Edge Geoserver transmits a DENM towards OBU 2 with the information of the event reported by OBU 1, and the warning is displayed on the web interface of OBU 2. Similarly, but in the opposite direction, OBU 1 is notified by the Edge Geoserver upon the reception of a DENM transmitted by OBU 2.

#### 5.2.2. MQTT-Based Communications

In this test case, both OBUs (1 and 2) use the MQTT protocol to communicate through the Edge MQTT broker. First, the MQTT Client of each OBU is connected to the Edge MQTT broker and both OBUs subscribe to the same topic that is associated with a geographical ROI. Subsequently, OBU 1 triggers the transmission of a DENM message towards the Edge MQTT broker, which subsequently distributes the DENM message to OBU 2. In the opposite direction, OBU 1 is notified by the Edge MQTT broker upon the reception of a DENM transmitted by OBU 2. The warnings are displayed on the web interface of each OBU. Listing 1 shows an extract of a JSON encode DENM message transmitted by one of the OBUs.

#### 5.2.3. Hybrid UDP/HTTP and MQTT-Based Communications

In this test case, OBU 1 communicates with the Edge Geoserver using UDP/HTTP protocol, and OBU 2 and the Edge Geoserver use the MQTT protocol to communicate via the Edge MQTT broker. The registration, authentication, and subscription to ROI steps of OBU 1 are identically performed, as described in test case 1. Similarly, the connection of OBU 2 to the Edge MQTT broker and the subscription of OBU 1 to a topic that is based on its location is performed, as described in test case 2. Subsequently, we trigger the transmission of a DENM message from OBU 1 towards the Edge Geoserver, and the Edge Geoserver publishes the DENM towards the Edge MQTT broker, which subsequently distributes the DENM message to OBU 2. In the opposite direction, OBU 2 triggers the transmission of a DENM message towards the Edge MQTT broker, and the Edge MQTT broker distributes the DENM message to the Edge Geoserver, which finally forwards the DENM message to OBU 1 over UDP.

### 5.3. Application-Level Latency Results

In this section, we experimentally evaluate the measured application-level latency of the ACCA service in order to demonstrate the effectiveness of interoperability in V2X communications using OBUs from different vehicle OEMs. The application-level latency is defined as the elapsed time since a DENM is transmitted by an OBU, or by the TMS, until the reception of that DENM by another OBU.

We have measured the application-level latency in two different scenarios based on the ACCA service architecture that is presented in Figure 1. In the first scenario, a road hazard is detected by one vehicle, and its OBU transmits a DENM message to the edge cloud application. In the second scenario, a road hazard is identified by the TMS, and it subsequently transmits a DENM message towards the edge cloud applications. It is worth noting that the TMS communicates with other traffic information sources outside the ACCA service; therefore, the TMS is capable of providing alerts on roadside hazardous events that are relevant to the connected vehicles within the ACCA service. Section 5.3.1 and Section 5.3.2, respectively, present the results for application-level latencies in both scenarios.

#### 5.3.1. Hazard Detected by a Vehicle

In this scenario, the application-level latency is measured by computing the elapsed time since the transmission of a DENM message by one of the OBUs, until subsequent reception of that DENM message on the other OBU. We have carried out two measurement campaigns. In the first campaign, OBU 1 and OBU 2 were connected to the same edge cloud and the V2X information exchange between both OBUs did not traverse through the TMS. In the second campaign, the OBUs were connected to two different edge clouds, with OBU 1 and OBU 2 being connected to edge cloud 1 and 2, respectively, establishing the communication between both edge clouds through the TMS, as shown in Figure 1.

For the connectivity of OBU 1 and OBU 2 with the edge clouds in each measurement campaign, we have considered three different combinations of V2X communication protocols that are described in Section 5.2: UDP/HTTP-based communications (Section 5.2.1); MQTT-based communications (Section 5.2.2); and, hybrid UDP/HTTP and MQTT-based communications (Section 5.2.3).

For the acquisition of latency results for each combination of V2X communication protocols in each measurement campaign, OBU 1 was first assigned the role of the sender and periodically generated 100 DENM messages, while OBU 2 was acting as the receiver, then, the roles of the OBUs were swapped and another 100 DENM messages were transmitted from OBU 2 to OBU 1. The time interval between consecutive DENM messages follows a uniform distribution between 3 and 5 s. In this way, we have collected 200 samples in total at each test case for the computation of the application layer latency.

**Figure 8:**
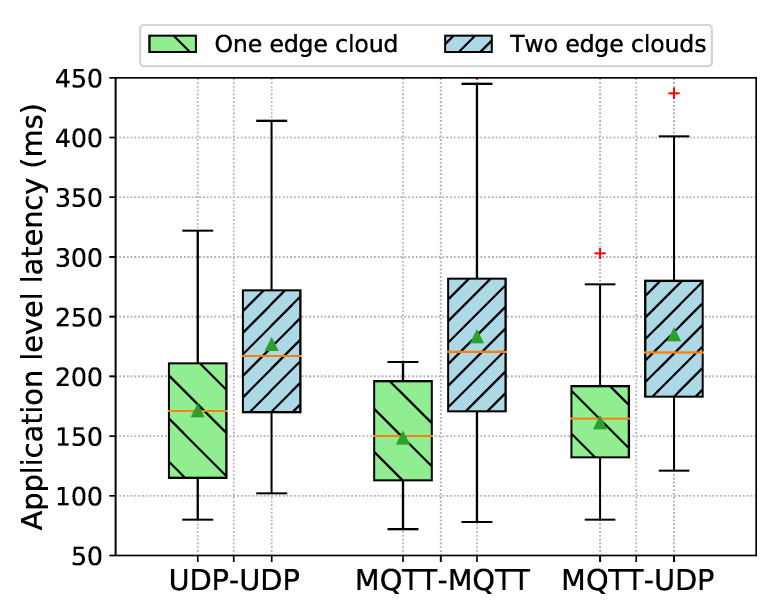
Application-level latency between OBUs measured for three different combinations of V2X communication protocols between the sender and the receiver OBUs. One edge cloud represents that both OBUs connected to the same edge cloud, whereas two edge clouds represent that each OBU is connected to a different edge cloud.

Figure 8 shows the results of application-level latency between the OBUs obtained in each measurement campaign (i.e., one edge cloud, two edge clouds). Each boxplot in the figure represents the distribution of measured application-level latencies over the total number of collected samples, indicating mean and median values, respectively, by the triangle and the horizontal line. The x-axis label indicates the combination of V2X communication protocols between the sending and the receiving OBUs, i.e., UDP-UDP indicates that both OBUs communicate over the UDP/HTTP protocol, MQTT-MQTT indicates that both OBUs communicate over MQTT, and UDP-MQTT indicates that OBU 1 communicates over UDP/HTTP and OBU 2 communicates over MQTT.

(1) Results of application-level latency for one edge cloud 

As it can be observed in Figure 8, when both OBUs are connected to the same edge cloud, the average application-level latency is: 171.0 ms with a standard deviation of 64.7 ms when both OBUs communicate over the UDP/HTTP protocol; 148.2 ms with a standard deviation of 45.0 ms when both OBUs communicate over MQTT; and, 161.1 ms with a standard deviation of 44.0 ms when OBU 1 communicates over UDP/HTTP and OBU 2 communicates over MQTT.

We have performed an ICMP-based PING on the OBUs to measure the Round Trip Time (RTT) between the OBUs and the edge cloud server over the 4G radio access network (RAN)in order to understand how much 4G/LTE contributes to the end-to-end application-level latency. The average value of the RTT is around 74 ms, thus, this is effectively accounting for around 43%, 50%, and 46% of the average application-level latency of UDP/HTTP-, MQTT-, and hybrid UDP/HTTP and MQTT-based communications, respectively, when both of the OBUs are connected to the same edge cloud. Therefore, we can observe that the rest of the delay is introduced from internal processing across layers in the backend and OBU applications.

It can be noticed that UDP/HTTP-based communication incurs extra delay of 23 ms on average than MQTT-based communication on the same edge cloud deployment, realising that this is due to the relatively higher internal processing delays introduced by the Edge Geoserver. Additionally, the Edge Geoserver synchronously sends unicast UDP messages to each connected OBU on the downlink by mapping each subscription to a corresponding OBU connection; hence, this also introduces extra delay into the end-to-end delay when compared to the publish-subscribe model of MQTT protocol. On the other hand, hybrid UDP/HTTP and MQTT-based communication introduces extra delay of 13 ms than MQTT-based communication; therefore, this can be attributed to the added processing delay introduced by the Edge Geoserver on top of processing by the Edge MQTT broker as well as delays in using the MQTT QoS level 1 for asynchronous reliable delivery of messages between participants [35,36]. The choice of QoS in MQTT has effects in the performance of throughput and the message transmission latency. In the ACCA service deployment, the Edge Geoserver performs the translation of ETSI ITS messages in ASN.1 format to JSON encoding, and vice versa. Thus, this translation mechanism introduces extra delay when compared to the Edge MQTT broker.

(2) Results of application-level latency for two edge clouds 

As it can be observed in Figure 8, when OBU 1 and OBU 2 are connected to two different edge clouds, the average application-level latency is: 226.5 ms with a standard deviation of 73.2 ms when the OBUs communicate over the UDP/HTTP protocol; 233 ms with a standard deviation of 86.7 ms when the OBUs communicate over MQTT; and. 234.8 ms with a standard deviation of 71 ms when OBU 1 communicates over UDP/HTTP and OBU 2 communicates over MQTT.

Similarly to the one edge cloud setup, the delay introduced by the 4G radio access network is effectively accounting for around 33%, 32%, and 32% of the average application-level latency of UDP/HTTP-, MQTT-, and hybrid UDP/HTTP and MQTT-based communications, respectively. Additionally, the average RTT over the wire-line (10 Gbps optical fiber connection) between the edge cloud servers and the TMS on the centralised cloud is around 1.45 ms; thus, the delay that occurs in inter-edge communication is negligible. Contrary to one edge cloud, we can observe that the delay over access network is contributing equally into the end-to-end delay of each combination, resulting in similar average delays, overall, on the application-level latency. However, it can be noticed slightly higher delays of few milliseconds in both MQTT, and hybrid UDP/HTTP and MQTT-based communications than UDP/HTTP-based communication. This is due to the involvement of additional processing elements in the backend. For example, V2X messages are processed twice by two instances of the Edge Geoservers, and this is additional to the Edge MQTT brokers in the scenario where MQTT-MQTT communication occurs over two edge clouds deployments; thus, it introduces relatively higher application-level latency, as shown in Figure 8, than UDP-UDP communication.

#### 5.3.2. Hazard Reported by the Traffic Management System

In this scenario, the application-level latency is measured by computing the elapsed time since the transmission of a DENM message by the TMS until the subsequent reception of that DENM message on OBU 1 and OBU 2. We have carried out one measurement campaign with OBU 1 and OBU 2 connected to the same edge cloud. OBU 1 communicates with the Edge Geoserver using the UDP/HTTP protocol, whilst OBU 2 uses the MQTT protocol to communicate via the Edge MQTT broker. Both of the OBUs were assigned the role of roadside hazardous events receivers reported from the TMS, which periodically transmitted a DENM message for a road incident at a fixed time interval of 10 s.

**Figure 9:**
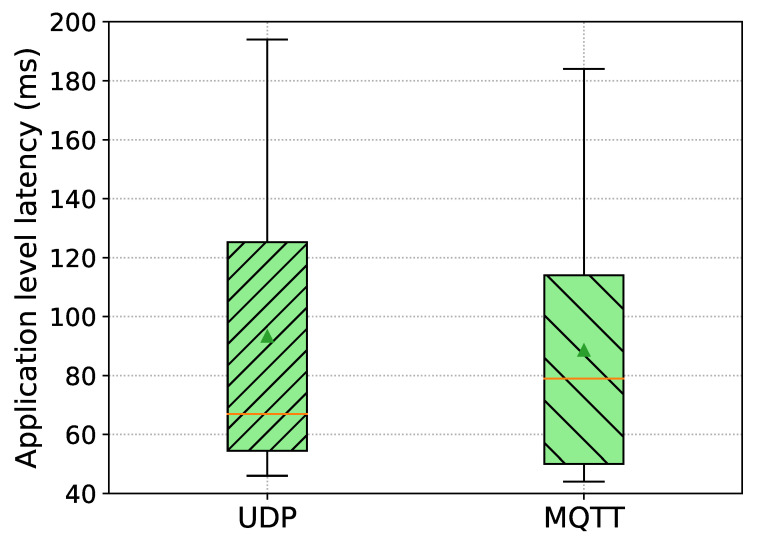
Application-level latency from the Traffic Management System (TMS) to the OBUs connected to the same edge cloud. OBU 1 receives DENM messages over UDP and OBU 2 receives over MQTT.

Figure 9 presented the results of application-level latency from the TMS to the OBUs. Each boxplot in the figure represents the distribution of measured application-level latencies over the total number of collected samples for each receiving OBU, indicating mean and median values, respectively, by the triangle and the horizontal line.

The average application-level latency from the TMS to the UDP/HTTP-based OBU is 93.3 ms with a standard deviation of 48.7 ms, and the average latency from the TMS to the MQTT-based OBU is 88.6 ms with a standard deviation of 47.6 ms, as can be observed in Figure 9. We devise the similar observations as was before such that effectively the delay (i.e., RTT/2) over the 4G radio access network is accounting for around 40% and 42% of the average application-level latency of UDP/HTTP- and MQTT-based communications, respectively. Moreover, it can be noticed that UDP/HTTP-based communication introduces an extra delay of close to 5 ms higher than MQTT-based communication. Similarly, we can make the same proposition as before, such that the Edge Geoserver introduces extra processing delays and, consequently, impacts on the application-level latency.

## 6. Discussion

The reported application-level latency results illustrated in Section 5.3 show the effectiveness of interoperability in V2X communication between different vehicle OEMs and, even more, it shows that our experimental OBU is functionally correct and it can be used to evaluate standards-compliant heterogeneous V2X communication architectures being interoperable with commercial OBUs. Furthermore, the ACCA service exposes widely used client-server (UDP/HTTP) and client-broker (MQTT) communication architectures, with request-response and publish-subscribe models, respectively, enabling seamless interoperability between the ACCA architecture and other similar systems. In turn, it extends the OBU’s capability of interworking with C-ITS systems outside ACCA, in which this has already confirmed by the reported latency results shown in Section 5.3.2. Overall, the obtained latency results highlight the tangible performance benefits when leveraging the edge-based service provisioning for V2X services, like the ACCA.

The data traffic in the ACCA service architecture is composed of small-sized messages transmission, and this does not impose strict bandwidth requirements on the communication links. Note that a single DENM message was in the order of 100 to 200 bytes, as there were no security-related components included. The frequency of DENM message generation in the tests was low when compared to CAM frequency. The message reception reliability measured at 100% without any packet drops in all test scenarios. Additionally, both of the radio links and wire-line between the edge cloud servers and the centralised cloud computing platform of the ACCA service deployment have not been congested during the tests from any other competing traffic in background.

## 7. Conclusions and Future Work

In this work, we have developed and functionally validated an experimental vehicular on-board unit (OBU) based on an open-source software implementation of the ETSI C-ITS protocol stack. Moreover, we have used the OBU to experimentally evaluate the application-level latency for V2X information exchange within an anticipated cooperative collision avoidance (ACCA) service while demonstrating the effectiveness of interoperability in V2X communication between OBUs from different vehicle original equipment manufacturers (OEMs). Our experimental OBU can readily be integrated into any vehicle, and it can use as a driver-assistance system in collision avoidance use cases. The OBU offers the flexibility to inter-work between different V2X protocols to seamlessly communicate with heterogeneous edge-/cloud-hosted services provided by different vehicle OEMs and Mobile Network Operators (MNOs). As future work, a field trial of the ACCA service infrastructure will be carried out in the city of Barcelona using a deployment of LTE Small Cells at 3.5 GHz and with edge cloud servers located in the street cabinet to evaluate key performance metrics under real traffic conditions. Through this field trial, we plan to evaluate the performance of the OBU in terms of latency and throughput, integrate security for secure V2X communications, and extend in-vehicle capabilities with smart sensors for hazards detection. In addition, we plan to use a performance monitoring platform that is based on Elasticsearch and Kibana that will allow the evaluation of data dissemination performance, such as network-level and application-level reliability. Moreover, we plan to leverage existing hardware-in-the-loop (HIL) simulation platforms with our OBU in order to evaluate communication delays under high density and high mobility road traffic scenarios for different radio access technologies.

## Data Availability

Not applicable.

## Abbreviations

The following abbreviations are used in this manuscript:

ACCA	Anticipated Cooperative Collision Avoidance
ASN.1	Abstract Syntax Notation One
BTP	Basic Transport Protocol
CAM	Cooperative Awareness Message
CAN	Control Area Network
CCAM	Cooperative, Connected and Automated Mobility
C-ITS	Cooperative Intelligent Transport System
C-V2X	Cellular Vehicle-to-Everything
DENM	Decentralized Environmental Notification Message
DSRC	Dedicated Short-Range Communications
ETSI	European Telecommunications Standards Institute
HMI	Human-Machine Interface
ITS	Intelligent Transport System
MQTT	Message Queuing Telemetry Transport protocol
MEC	Mobile Edge Computing
MNO	Mobile Network Operator
OBU	On-Board Unit
OEM	Original Equipment Manufacturer
RAN	Radio Access Network
ROI	Region of Interest
RSU	Roadside Unit
TMS	Traffic Management System
V2I	Vehicle-to-Infrastructure
V2N	Vehicle-to-Network
V2P	Vehicle-to-Pedestrian
V2V	Vehicle-to-Vehicle
V2X	Vehicle-to-Everything
